# Cryptic lineages hybridize for worker production in the harvester ant *Messor barbarus*

**DOI:** 10.1098/rsbl.2016.0542

**Published:** 2016-11

**Authors:** Victoria Norman, Hugo Darras, Christopher Tranter, Serge Aron, William O. H. Hughes

**Affiliations:** 1 School of Life Sciences, University of Sussex, Brighton, East Sussex BN1 9QG, UK; 2 Evolutionary Biology and Ecology, Université Libre de Bruxelles, Avenue Franklin D. Roosevelt, 50, CP 160/12, Brussels 1050, Belgium

**Keywords:** hybridization, social insect, caste determination, social hybridogenesis

## Abstract

The reproductive division of labour between queen and worker castes in social insects is a defining characteristic of eusociality and a classic example of phenotypic plasticity. Whether social insect larvae develop into queens or workers has long been thought to be determined by environmental cues, i.e. larvae are developmentally totipotent. Contrary to this paradigm, several recent studies have revealed that caste is determined by genotype in some ant species, but whether this is restricted to just a few exceptional species is still unclear. Here, we show that the Mediterranean harvester ant *Messor barbarus* possesses an unusual reproductive system, in which the female castes are genetically determined. Using both nuclear and mitochondrial data, we show that Iberian populations have two distinct, cryptic lineages. Workers are always inter-lineage hybrids whereas queens are always produced from pure-lineage matings. The results suggest that genetic caste determination may be more widespread in ants than previously thought, and that further investigation in other species is needed to understand the frequency and evolution of this remarkable reproductive system.

## 1. Introduction

Phenotypic plasticity is a widespread and fundamental trait that enables organisms to adapt their phenotype during development to prevailing environmental conditions [1]. One of the classic examples of phenotypic plasticity is the morphological castes exhibited by social insects. The reproductive division of labour between reproductive queens and non-reproductive workers is arguably the defining trait of the major evolutionary transition to eusociality [2], and the caste destiny of a developing individual determines whether it will achieve fitness directly through its own reproduction or indirectly by enhancing the reproduction of its relatives.

The development of a diploid egg into a reproductive queen or non-reproductive worker in social Hymenoptera (ants, some bees and some wasps) is generally thought to be determined by environmental cues, with larvae being developmentally totipotent with respect to their caste fate. However, a number of cases of genotypic influences on caste determination have now been reported [3], the most extreme of which are a small number of ant species that exhibit social hybridogenesis [4–10]. This remarkable reproductive system involves workers being produced sexually from matings between two lineages or even species, and queens being produced exclusively either from within-lineage matings or in some species by parthenogenesis. Social hybridogenesis is generally detected by microsatellite genotyping of the worker and new queen (gyne) offspring in colonies, with the genotypes of workers indicating that their parents were from two distinct lineages whereas the genotypes of gynes indicate both their parents were from the same lineage [4–9].

Social hybridogenesis has fundamental implications for caste determination because hybrid females are constrained to become workers. This leads to a very strong association between genetic material and reproductive potential, and therefore subsequent genetic caste determination. Social hybridogenesis in social insects is consequently thought to be a rare and exceptional phenomenon [4–9]. Here, we describe a new case of social hybridogenesis, the Mediterranean harvester ant *Messor barbarus*.

## 2. Material and methods

**Figure 1.**
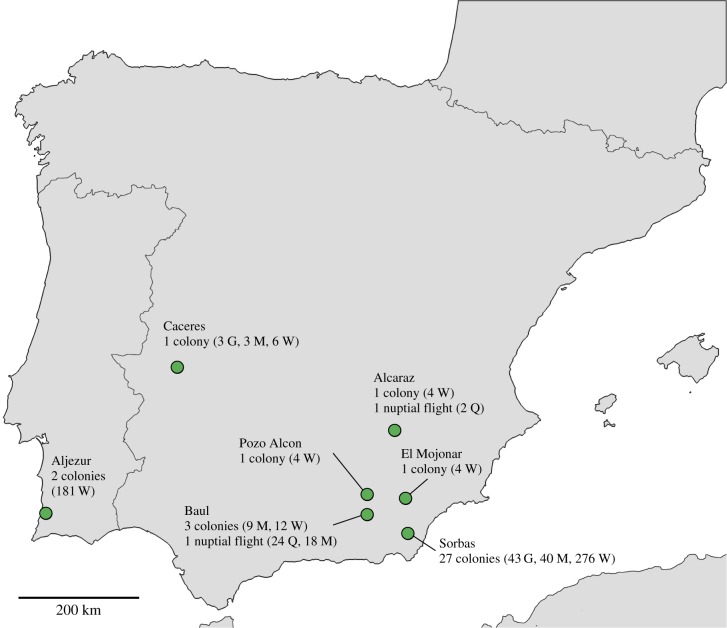
Sampling locations of *Messor barbarus* harvester ants in the southern Iberian Peninsula (number of colonies excavated and number of individuals genotyped; Q, mated queens; G, virgin queens; M, males; W, workers). (Online version in colour.)

The harvester ant *M. barbarus* is a common species in the dry habitat of the Iberian Peninsula. Colonies contain a single queen and several thousand workers. We collected workers, and gynes (new queens) and males when present, from 27 colonies in Sorbas (Spain) and nine additional colonies in Baul, Pozo Alcón, El Mojonar, Alcaraz, Cáceres (Spain) and Aljezur (Portugal), and queens and males during nuptial flights in Baul and Alcaraz (figure 1). We used mitochondrial DNA and nuclear microsatellites to infer population and colony structure. We sequenced a portion of the COI mtDNA gene for 37 individuals from the seven sampling sites (one individual from each of 28 colonies covering all sites apart from Baul, plus 1 queen from the Alcaraz nuptial flight and 8 queens from the Baul nuptial flight), and constructed maximum-likelihood trees. We genotyped a selection of workers, gynes and males from the 36 colonies (590 ants in total), using four polymorphic microsatellite markers (*Ms1a*, *Ms2a*, *Ms2c* and *Ms2d*; [11]) and inferred the genotypes of the mother queen and her mates for each colony from the multi-locus microsatellite genotypes (non-detection error of *P_Mbar1_* = 0.0086 and *P_Mbar2_* = 0.0030; see the electronic supplementary material). We calculated the effective mating frequencies of mother queens using an estimator that takes into account sample size (we did this separately for worker and gyne offspring because they were produced by different types of mating; see Results and electronic supplementary material). We determined population structure using the 158 reproductive individuals in our sample for which we had microsatellite genotypes (inferred parental genotypes of 30 colonies, and 26 queens and 18 males from the two nuptial flights) with structure and genetic-distance-based PCoA. To explore mating patterns, we augmented the PCoA with connections between parental genotypes found co-occurring in the offspring. See the electronic supplementary material for full methods.

## 3. Results

**Figure 2.**
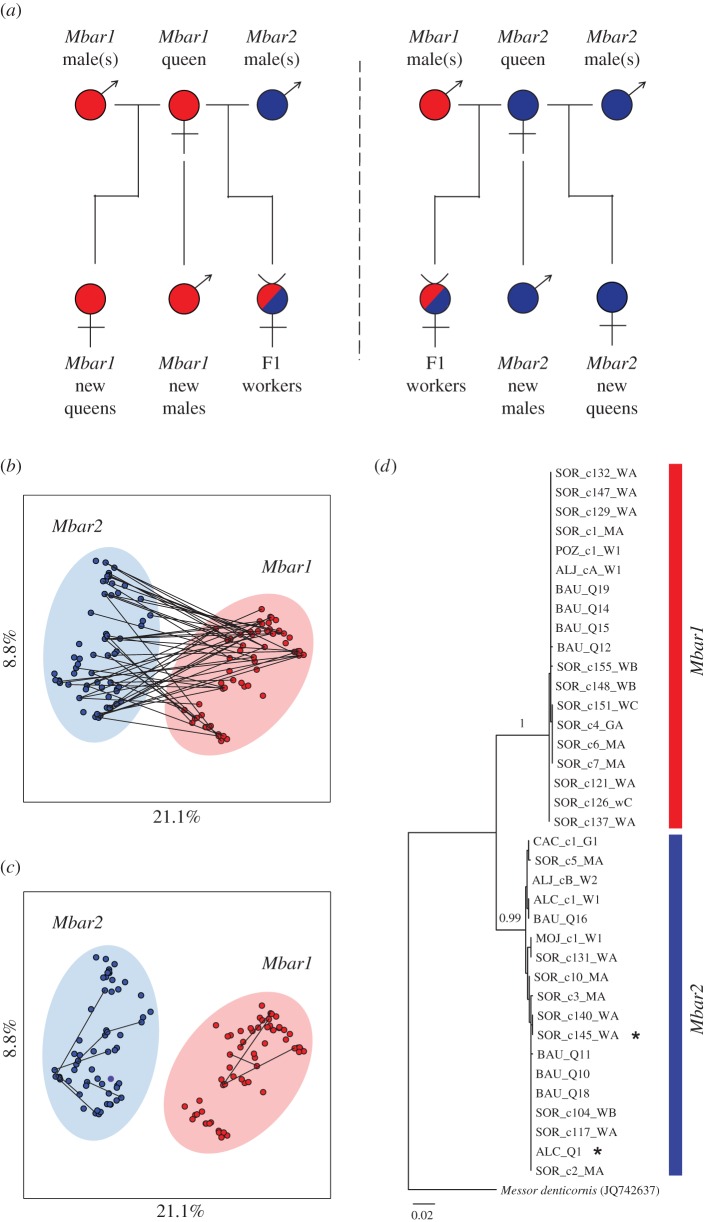
Social hybridogenesis in *Messor barbarus* harvester ants in which two genetic lineages (*Mbar1* and *Mbar2*) coexist within the population. (*a*) Pure-lineage matings produce queens whereas inter-lineage matings always produce sterile workers. (*b*,*c*) PCoA plot based on genotypes of 129 reproductive individuals (nuclear DNA) augmented with connections between parental genotypes found co-occurring in (*b*) workers and (*c*) gynes (new queens). The percentage of variation explained by each PCoA axis is indicated. (*d*) Maximum-likelihood tree inferred from a portion of COI gene (mitochondrial DNA). Numbers at nodes indicate bootstrap values. Specimen name gives population (SOR: Sorbas, BAU: Baul, POZ: Pozo Alcón, MOJ: El Mojonar, ALC: Alcaraz, CAC: Cáceres, ALJ: Aljezur), colony number and caste. The two individuals with a star symbol are cyto-nuclear mismatches. (Online version in colour.)

Mitochondrial haplotypes clustered into two groups (*Mbar1* and *Mbar 2*; figure 2*d*). Both were found in Sorbas, Baul and Aljezur, suggesting that the two groups were sympatric across the studied area. In the Baul nuptial flight, 20 sexuals were *Mbar1* and 22 were *Mbar2*, while in Sorbas the mother queens of nine colonies were *Mbar1* and of seven colonies *Mbar2*, so in both cases an approximately equal ratio. Offspring microsatellite genotypes enabled us to infer the genotype of the mother queen for 30/36 colonies, and paternal genotypes for 29 of these (electronic supplementary material, table S1; [12]). The genotype data for each colony were consistent with individuals being the offspring of a single queen that had been inseminated by one or multiple males (gynes and workers were produced by different types of mating and gynes were only available for a subset of colonies, so values here are based on worker genotypes only: mean ± s.e. observed mating frequency *M*_obs_ = 2.41 ± 0.16; effective mating frequency *M*_eff_ = 2.24 ± 0.16 using all 27 colonies or *M*_eff_ = 2.36 ± 0.22 using only the 18 colonies for which at least eight workers were genotyped). Based on worker genotypes, *Mbar1* queens had slightly lower effective mating frequencies than *Mbar2* queens (see the electronic supplementary material). All workers (*N* = 491) and new queens (*N* = 47) were found to be produced by sexual reproduction, whereas all males were haploid (*N* = 52).

Bayesian clustering of the microsatellite genotypes of reproductive individuals suggested the presence of two groups, one that always had allele 166 at *Ms2c*, and a second that never had allele 166 (see the electronic supplementary material; *N* = 158 reproductive individuals, for *N* = 129 of which *Ms2c* genotypes were successfully determined). These two groups were congruent with the mtDNA *Mbar1* and *Mbar2* lineages, with the exception of two individuals (the ALC_Q1 queen and the inferred mother queen of worker SOR_c145_WA) that had the *Mbar2* mitochondrial haplotype but *Mbar1* microsatellite genotypes (figure 2*d*). The *Mbar1* and *Mbar2* lineages were highly differentiated (*F*_ST_ = 0.28) at three microsatellite loci (*Ms2c*, *Ms2a* and *Ms2d*; *p* < 0.001 in each case), and also significantly differentiated at the fourth (*Ms1a*; *p* = 0.029). Each lineage occupied a distinct area of the PCoA plot (figure 2*b*,*c*). Remarkably, all gynes (new queens) were found to arise from within-lineage matings (*N* = 47, figure 2*c*), while all workers were found to arise from inter-lineage matings (*N* = 491; figure 2*b*). In line with this, the 276 workers genotyped from 22 colonies from Sorbas were more heterozygous than expected under Hardy–Weinberg equilibrium (*F*_IS_ = −0.18, *p* < 0.0001). In all populations, workers were heterozygous at *Ms2c* with allele 166 and a different allele.

## 4. Discussion

Our genetic analyses based on mitochondrial DNA and nuclear DNA revealed the existence of an unusual population structure in the harvester ant *M. barbarus*. Two cryptic lineages (*Mbar1* or *Mbar2)* coexist in Iberian populations. These lineages appear to be inter-dependent because all workers were found to be produced from inter-lineage mating. Despite constant hybridization for hybrid worker production, the lineages are consistently genetically divergent across generations: haploid males are produced asexually and diploid queens are produced by same-lineage mating (figure 2*a*). Two queens had cyto-nuclear mismatches (mtDNA from one lineage and nDNA from the other one), suggesting that rare introgression events may exist (see [13] for potential mechanisms). Applying molecular clocks of 2.3–4% per million years (standard clock and upper bound estimate for insect COI; [14]), the divergence of *Mbar1* and *Mbar2* mtDNA was estimated to be 1.4–2.5 million years. The system itself may, however, be much more recent as the two lineages could have arisen from secondary contact between previously geographically isolated populations or from two different species.

In most ant species, the development of a diploid egg into a reproductive queen or a non-reproductive worker is thought to be determined by environmental cues, although genotypic influences on caste determination have been reported [3]. In *M. barbarus*, the caste-genotype association suggests the existence of strong genotypic effects. Such reduced totipotency of hybrid (worker) and non-hybrid (sexual) brood requires queens to mate multiply with males of both lineages to produce a viable colony and achieve reproductive fitness [15]. Remarkably, however, observed mating frequencies in *M. barbarus* were found to be low enough that a large proportion of queens (approx. 13.4%; see the electronic supplementary material) would fail to mate with both males of their own lineage and those of the other lineage, and therefore be either unable to produce the workers necessary for a viable colony or unable to produce gyne offspring, if mating is random. Inter-lineage mating pairs (based on offspring genotypes) were more numerous than same-lineage pairs (see the electronic supplementary material), suggesting that mating may be non-random and that there is some pre-zygotic sexual selection, something that is relatively unknown in social insects. Nevertheless, there seems likely to be a significant fitness cost associated with the system, implying that there must also be a significant fitness advantage associated with social hybridogenesis for the phenomenon to have evolved. Hybrid vigour of workers is one possibility [7].

The reproductive system of *M. barbarus* is strikingly similar to that of *Pogonomyrmex* harvester ants, in which inter-dependent lineage pairs also occur [16]. These lineages have been shown to harbour chimaeric genomes of two parental species, but the causal link between this hybrid origin and the actual system remains controversial [17]. Lineages of *M. barbarus* may also be of interspecific hybrid origin as unusual colony mixing and hybridization has been previously reported in the genus [18].

Remarkably, social hybridogenesis with a variety of mechanisms has now been reported in seven phylogenetically widespread ant genera: five myrmicines (*Messor*, *Pogonomyrmex*, *Solenopsis*, *Vollenhovia* and *Wasmania*), and two formicines (*Cataglyphis* and *Paratrechina*) [10]. The multiple independent origins of these systems suggest a general predisposition for the evolution of hybrid workers in ants, but the exact mechanism(s) have remained difficult to untangle. It is notable that three of the cases of social hybridogenesis involve species in arid environments (*Messor*, *Pogonomyrmex* and *Cataglyphis*), and two involve invasive ants (*Wasmania* and *Paratrechina*; *Volenhovia emeryi* is also an invasive species, though not, it is thought, in the area in which genetic caste determination has been described), suggesting that life-history characteristics may select for the evolution of this remarkable reproductive strategy. Hopefully, the accumulation of genomic data for more ant species will provide some clues to how these unorthodox reproductive systems originate.

## Supplementary Material

Supplementary methods and results

## Data accessibility

DNA sequences were deposited in GenBank KX698041-KX698077. Microsatellite genotype data are available in Dryad: http://dx.doi.org/10.5061/dryad.pq373 [12].
