# Supplementary material for: Cryptic lineages hybridize for worker production in the harvester ant Messor barbarus
Source: Biol Lett. 2016 Nov;12(11):20160542. doi: 10.1098/rsbl.2016.0542 (PMC5134035; doi:10.1098/rsbl.2016.0542)

**Electronic Supplementary Material**

**Materials and methods**

The harvester ant *Messor barbarus* is a common species in the dry habitat of the Iberian Peninsula. Colonies contain a single queen and several thousand workers. We excavated 27 partial colonies in Sorbas (Spain) and nine additional colonies in Baul, Pozo Alcón, El Mojonar, Alcaraz, Cáceres (Spain) and Aljezur (Portugal). Samples of workers and, when present, gynes (new queens) and males, were collected for subsequent genetic analyses. We also sampled reproductives (queens and males) during nuptial flights in Baul and Alcaraz (figure 1). We used mitochondrial DNA sequence data and microsatellite markers to infer both population and colony structure across our sampling. DNA was extracted from adult ants with Chelex-100.

For the analysis of mtDNA, a portion of COI was amplified for 37 individuals from the seven sampling sites: one individual from each of 28 colonies covering all sites apart from Baul, plus 1 gyne from the Alcaraz nuptial flight and 8 gynes from the Baul nuptial flight. We amplified COI using the universal primers HCO2198 and LCO1490 [1], and an annealing temperature of 50°C. PCR products were sequenced from both ends. Sequences were checked for quality, aligned and trimmed using CodonCode Aligner 4.1. Maximum likelihood trees were constructed with MEGA7 [2]. JModelTest 2 [3] suggested GTR + I + G as the best substitution model based on AIC criterion. Branch support values were obtained by 1000 bootstrap pseudo-replicates. A published sequence of *Messor denticornis* was used as an outgroup (GenBank: JQ742637).

Nuclear DNA variation and pedigree were studied using 4 microsatellite markers (*Ms1a*, *Ms2a*, *Ms2c* and *Ms2d*; [4]). All gynes and males as well as selection of workers were genotyped. For each colony, the genotypes of the mother queen and her mates were inferred from workers whenever possible. The effective mating frequencies of queens were estimated from worker genotypes using the bias-corrected estimator of effective number of mates (M_ep_) proposed by Nielsen et al. [5]. Although this estimator takes into account sample size, we also estimated effective mating frequencies both including all colonies and using only colonies for which at least 8 workers were genotyped. Because gynes and workers were found to be produced by different types of mating (see results), we estimated the effective mating frequency of queens both based on worker offspring (M_epW_) and based on gyne offspring (M_epG_) for those colonies for which both were available. To verify the reliability of our four microsatellite markers to detect different patrilines, we first calculated the probability that two random fathers shared the same alleles at all loci [9]. In Sorbas, where we had good estimates of allele size frequencies, the non-detection error due to two males sharing the same alleles was very low for both lineages (P*_Mbar1_*=0.0086 and P*_Mbar2_*=0.0030). *F*-statistics were estimated using SPAGeDi [6]. Observed values were compared with the corresponding frequency distributions when random permutations of the data were performed. To determine the population structure, we excluded all worker genotypes and used inferred parents only. Haploid male genotypes were encoded as diploid by doubling their alleles. The number of groups (K) among reproductives was determined using STRUCTURE [7]. The program was run 10 times for each value of K = 1–10 under an admixture model with correlated allele frequencies, with 100,000 Markov chain Monte Carlo iterations and a burn-in period of 20,000. The number of group was investigated using the *ad hoc* d-K method [8]. For cross-validation of STRUCTURE results, a genetic-distance-based PCoA was performed with GENALEX v6.5. To explore mating patterns, we augmented the PCoA with connections between parental genotypes found co-occurring in the offspring.

**Results**

Mitochondrial COI sequences were trimmed to 669 base pairs, among which 45 (6.73 %) were variable. Haplotypes clustered in two groups (figure 2d) separated by an average of 38.2 base pairs (5.71%). The two groups will be hereafter referred as *Mbar1* and *Mbar2*. Pairwise sequence divergences within each group were comparatively low: 0.48% and 0.12%, respectively. Both *Mbar1* and *Mbar2* genotypes were found in Sorbas, Baul and Aljezur, suggesting that the two groups were sympatric across the studied area.

We genotyped 634 ants at four microsatellite loci (590 ants from the 36 colonies, plus 44 sexuals from the two nuptial flights; 2.9% missing data). We were able to infer the genotype of the mother queen for 30/36 colonies, and paternal genotypes for 29/36 colonies. All colonies were consistent with there being a single mother who has been inseminated by one or multiple mates (see later). There was a significant genetic differentiation between colonies, confirming that all colonies were genuine and that there were no cases of polydomy in the data (global-F_ST_ = 0.2386, test by random permutations of individual among pairs of colonies: P < 0.05 for all colony pairs). As is usually the case in Hymenoptera, all workers and new queens had genotypes at one or more loci that were non-identical to their mother and therefore had been produced by sexual reproduction (N = 491 and N = 47, respectively), whereas males were haploid individuals produced by arrhenotokous parthenogenesis (N = 52, figure 1).

Bayesian clustering analysis using STRUCTURE of reproductive individuals (inferred parental genotypes of colonies, and queens and males from the two nuptial flights; N = 158) suggested the presence of two groups. The two groups could be diagnosed based on their genotype at *Ms2c*. The first group consists of reproductive individuals with allele 166 (or rare allele 168) at *Ms2c*, whereas the second group had alleles bigger than 166 only. For 29 individuals, we had ambiguous genotype information at *Ms2c*. Yet, these were consistent with having either allele 166 or alleles bigger than 166 only. We did not find any heterozygous queens with both the allele 166 (or 168) plus a bigger allele. We used the 129 individuals with unambiguous genotype information at *Ms2c* for the following analyses. The two groups were congruent with the *Mbar1* and *Mbar2* lineages observed based on mitochondrial DNA with the exception of two individuals (figure 2d). One of the two exceptions was a queen (ALC_Q1) with the *Mbar2* mitochondrial haplotype but *Mbar1* microsatellite genotype. This may be a hybrid queen, but additional markers would be required to confirm this. The other exception was a worker (SOR_c145_WA) with the *Mbar2* mitochondrial haplotype but an inferred mother with *Mbar1* microsatellite genotype (the worker herself was, as with all workers, a hybrid). This could be explained by the mother queen being a hybrid or by paternal leakage of mitochondrial DNA.

The two lineages were highly differentiated (F_ST_ = 0.28) at three microsatellite markers (*Ms2c*, *Ms2a* and *Ms2d*; test by random permutations of individual among lineages: P < 0.001 for each marker), and marginally differentiated at the fourth (*Ms1a*; P = 0.029). Each lineage occupied a distinct area of the PCoA plot. Remarkably, all gynes were found to arise from within-group mating (N = 47, figure 2c), while all workers were found to be inter-lineages individuals (figure 2b). In line with this, the 276 workers sampled in the 27 colonies from Sorbas were more heterozygous than expected under Hardy–Weinberg equilibrium (F_IS_ = -0.18, test by random permutations of allele sizes among among individuals: P < 0.001). In all populations, workers were heterozygous at Ms2c with allele 166 (or 168) plus an allele bigger than 166.

The overall mean effective mating frequency of *M. barbarus* based on worker genotypes (M_epW_) was 2.24 ± 0.16 using all colonies (N = 27 colonies), or 2.36 ± 0.22 using only colonies for which at least 8 workers were genotyped (N = 18 colonies). The effective mating frequencies based on worker genotypes of *Mbar1* queens were slightly smaller than for *Mbar2* queens (all colonies: *Mbar1* M_epW_ = 1.91± 0.2, N = 15 colonies and *Mbar2* M_epW_ = 2.71 ± 0.22, N = 11 colonies, *t* = 2.62, df = 24, P = 0.015; colonies for which at least 8 workers genotyped: *Mbar1* M_epW_ = 2.03 ± 0.25, N = 11 colonies and *Mbar2* M_epW_ = 3.01 ± 0.36, N = 6 colonies, *t* = 2.3, df = 15, P = 0.036). Intriguingly, the effective mating frequencies of mother queens estimated from their gyne offspring (M_epG_) appeared significantly smaller than when estimated from their worker offspring (based on colonies for which we had both M_epW_ and M_epG_; overall: M_epG_ = 1.3 vs. M_epW_ = 2.3, N = 6 colonies of the two lineages, *t* = 2.61, df = 5, P = 0.048; for *Mbar2* lineage only: M_epG_ = 1.36 vs. M_epW_ = 2.56, N = 5 colonies, *t* = 2.99, df = 4, P = 0.04). However, as this is based on only six colonies, five of which were *Mbar2*, and for five of which the numbers of workers or gynes genotyped were small (< 8), it will need further research to determine if this is a genuine difference.

Assuming an equal frequency of *Mbar1* and *Mbar2* in a population (as was found at Sorbas and Baul) and a total effective queen mating frequency (M_obW_ + M_obG_) of 2.41 + 1.5 = 3.91, the likelihood of a queen of one lineage failing to mate with at least one male of the other lineage (and therefore being unable to produce the workers necessary for a viable colony) can be estimated as 0.5^3.91^ = 6.7%. The probability of a queen failing to mate with at least one male of her own lineage (and therefore being unable to produce gyne offspring) is similarly 6.7%. The combined probability of a queen failing to obtain the necessary matings to both produce the workers necessary for a viable colony and produce gynes is then 13.4%.

**References**

1. Folmer, O., Black, M., Hoeh, W., Lutz, R. & Vrijenhoek, R. 1994 DNA primers for amplification of mitochondrial cytochrome c oxidase subunit I from diverse metazoan invertebrates. *Mol. Mar. Biol. Biotechnol.* **3**, 294–9.

2. Kumar, S., Stecher, G. & Tamura, K. 2016 MEGA7: Molecular Evolutionary Genetics Analysis version 7.0 for bigger datasets. *Mol. Biol. Evol.* , 1–11. (doi:10.1093/molbev/msw054)

3. Darriba, D., Taboada, G. L., Doallo, R. & Posada, D. 2012 jModelTest 2: more models, new heuristics and parallel computing. *Nat. Methods* **9**, 772. (doi:10.1038/nmeth.2109)

4. Arthofer, W., Schlick-Steiner, B. C., Steiner, F. M., Konrad, H., Espadaler, X. & Stauffer, C. 2005 Isolation of polymorphic microsatellite loci for the study of habitat fragmentation in the harvester ant *Messor structor*. *Conserv. Genet.* **6**, 859–861. (doi:10.1007/s10592-005-9033-5)

5. Nielsen, R., Tarpy, D. R. & Reeve, H. K. 2003 Estimating effective paternity number in social insects and the effective number of alleles in a population. *Mol. Ecol.* **12**, 3157–64.

6. Hardy, O. J. & Vekemans, X. 2002 spagedi: a versatile computer program to analyse spatial genetic structure at the individual or population levels. *Mol. Ecol. Notes* , 618–620.

7. Pritchard, J. K., Stephens, M., Rosenberg, N. A. & Donnelly, P. 2000 Association mapping in structured populations. *Am. J. Hum. Genet.* **67**, 170–81. (doi:10.1086/302959)

8. Evanno, G., Regnaut, S. & Goudet, J. 2005 Detecting the number of clusters of individuals using the software STRUCTURE: a simulation study. *Mol. Ecol.* **14**, 2611–20. (doi:10.1111/j.1365-294X.2005.02553.x)

9. Boomsma, J.J. & Ratnieks, F.L.W. 1996 Paternity in eusocial Hymenoptera. *Phil. Trans. R. Soc. Lond. B.* **351**, 947-975.

**Figure S1.** PCoA plot based on the genotypes of 158 reproductive individuals (inferred parents of colonies, and queens and males collected during nuptial flights). The percentage of variation explained by each PCoA axis is indicated. *Mbar1*: red, *Mbar2*: blue, unknown: grey (missing information for *Ms2c*).

**Figure S2.** Structure bar plot showing the assignment probabilities for *K* = *2* of: a) the 158 reproductive individuals genotyped, including 29 individuals that could not be assigned to a lineage due to ambiguous information at *Ms2c*; b) the 129 reproductive individuals assigned to a lineage, c) the 129 reproductive individuals assigned to a lineage and also genotypes for 36 workers (one from each colony sampled). For each plot, the highest probability run is shown.

**Figure S3.** Allele frequencies for *Mbar1* (red bars) and *Mbar2* (blue bars) at the four microsatellite loci surveyed based on the genotypes of 129 reproductive individuals (inferred parents of colonies, and queens and males collected during nuptial flights) with known genotype information at Ms2c.

**Table S1**. Lineage, numbers of worker and gyne (new queens) offspring genotyped, and mating frequency estimates based on offspring genotypes, for 29 Iberian *Messor barbarus* colonies studied. Mating frequency estimates are observed (M_obs_) and effective (M_ep_) mating frequencies based on worker (_W_) or gyne (_G_) offspring. Colonies for which offspring genotypes did not allow parental genotypes to be determined are indicated by nd. The lineage of colonies SOR_c118 and SOR_c141 are unknown.

| **Colony** | **Lineage of mother queen** | **Number of offspring genotyped** | | | **# queens** | **Mating frequency estimates of mother queens** | | | |
| --- | --- | --- | --- | --- | --- | --- | --- | --- | --- |
|  |  | **workers** | **gynes** | **males** |  | **M_obsW_** | **M_obsG_** | **M_epW_** | **M_epG_** |
| ALJ_cA | *Mbar1* | 89 |  |  | 1 | 3 |  | 2.22 |  |
| ALJ_cB | *Mbar2* | 92 |  |  | 1 | 2 |  | 1.89 |  |
| ALC_c1 | *Mbar2* | 4 |  |  | nd | nd |  | nd |  |
| BAU_c1 | *Mbar1* | 4 |  | 4 | 1 | 1 |  | 1.00 |  |
| BAU_cA | *Mbar2* | 4 |  | 4 | 1 | 2 |  | 2.25 |  |
| BAU_cB | *Mbar1* | 4 |  | 1 | 1 | nd |  | nd |  |
| CAC_c1 | *Mbar2* | 6 | 3 | 3 | 1 | nd | 2 | nd | 1.80 |
| MOJ_c1 | *Mbar2* | 4 |  |  | nd | nd |  | nd |  |
| POZ_c1 | *Mbar1* | 8 |  |  | 1 | 3 |  | 3.61 |  |
| SOR_c1 | *Mbar1* | 6 | 8 | 1 | 1 | nd | 2 | nd | 1.32 |
| SOR_c2 | *Mbar2* | 5 | 2 | 4 | 1 | 2 | 1 | 2.25 | 1.00 |
| SOR_c3 | *Mbar2* | 5 | 6 | 4 | 1 | 2 | 2 | 2.17 | 1.99 |
| SOR_c4 | *Mbar1* | 5 | 10 |  | 1 | 1 | 1 | 1.00 | 1.00 |
| SOR_c5 | *Mbar2* | 6 | 3 | 3 | 1 | 3 | 2 | 2.27 | 1.80 |
| SOR_c6 | *Mbar1* | 5 |  | 6 | 1 | 2 |  | 2.17 |  |
| SOR_c7 | *Mbar1* | 5 |  | 6 | 1 | 2 |  | 2.17 |  |
| SOR_c8 | *Mbar2* | 9 | 1 | 5 | 1 | 4 | nd | 3.69 | nd |
| SOR_c9 | *Mbar1* | 9 | 2 |  | 1 | 1 | nd | 1.00 | nd |
| SOR_c10 | *Mbar2* | 9 | 9 | 4 | 1 | 3 | 1 | 3.36 | 1.00 |
| SOR_c11 | *Mbar2* | 7 | 3 | 7 | 1 | 3 | 1 | 2.74 | 1.00 |
| SOR_c104 | *Mbar2* | 13 |  |  | 1 | 4 |  | 2.82 |  |
| SOR_c117 | *Mbar2* | 13 |  |  | nd | nd |  | nd |  |
| SOR_c118 | nd | 14 |  |  | 1 | 2 |  | 2.09 |  |
| SOR_c121 | *Mbar1* | 15 |  |  | 1 | 2 |  | 1.89 |  |
| SOR_c126 | *Mbar1* | 14 |  |  | 1 | 3 |  | 3.17 |  |
| SOR_c129 | *Mbar1* | 11 |  |  | nd | nd |  | nd |  |
| SOR_c131 | *Mbar2* | 13 |  |  | 1 | 4 |  | 4.16 |  |
| SOR_c132 | *Mbar1* | 13 |  |  | 1 | 2 |  | 1.61 |  |
| SOR_c137 | *Mbar1* | 12 |  |  | 1 | 3 |  | 2.58 |  |
| SOR_c140 | *Mbar2* | 11 |  |  | 1 | 2 |  | 2.15 |  |
| SOR_c141 | nd | 13 |  |  | nd | nd |  | nd |  |
| SOR_c145 | *Mbar1* | 16 |  |  | 1 | 2 |  | 1.14 |  |
| SOR_c147 | *Mbar1* | 9 |  |  | 1 | 2 |  | 1.95 |  |
| SOR_c148 | *Mbar1* | 10 |  |  | nd | nd |  | nd |  |
| SOR_c151 | *Mbar1* | 15 |  |  | 1 | 3 |  | 1.56 |  |
| SOR_c155 | *Mbar1* | 13 |  |  | 1 | 2 |  | 1.61 |  |

**Fig. S1**


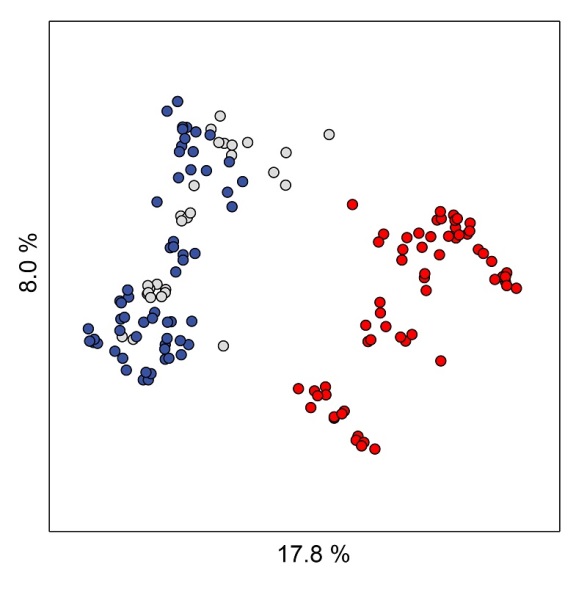


**Fig. S2**

**
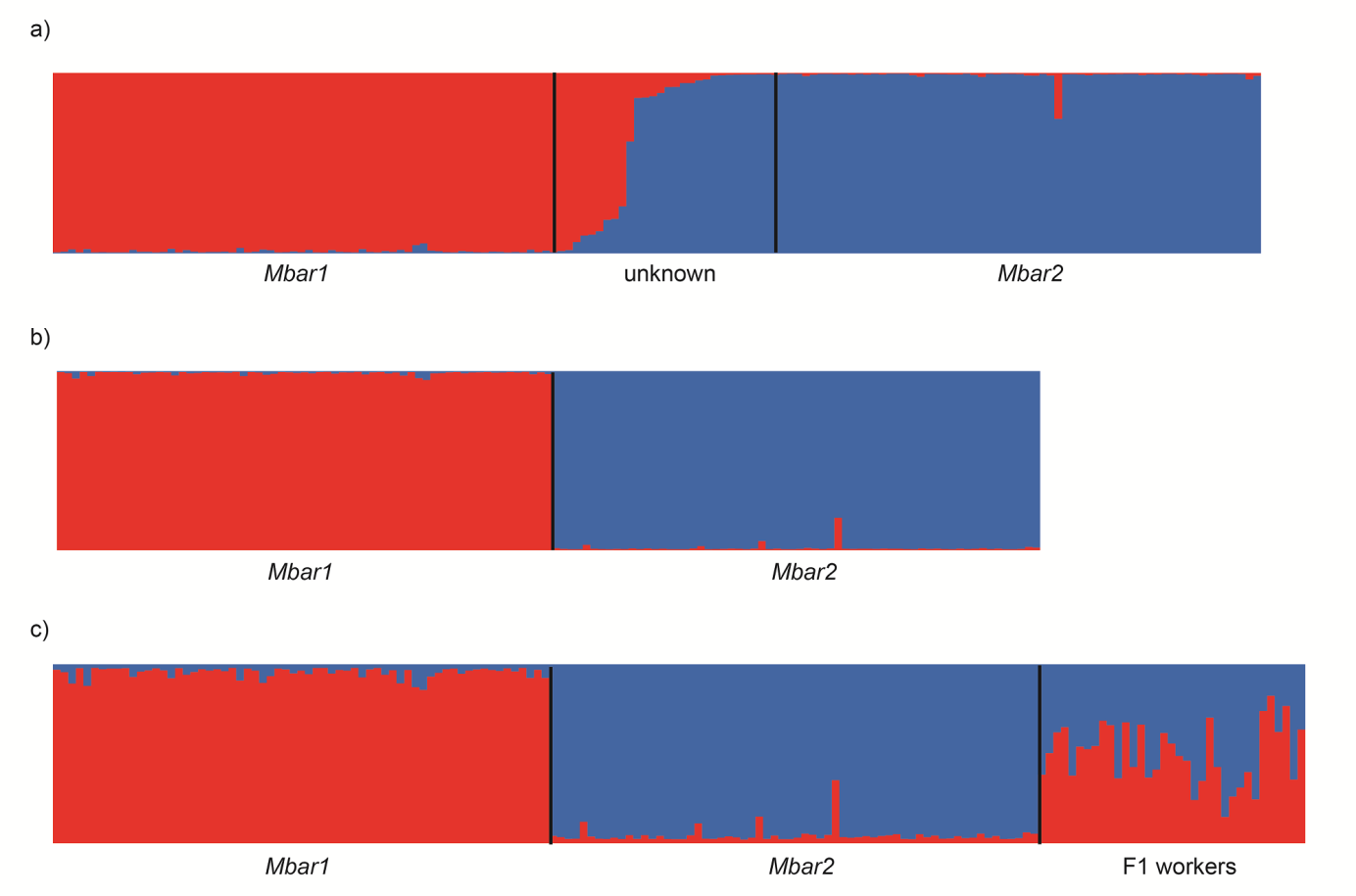
**

**Fig. S3**


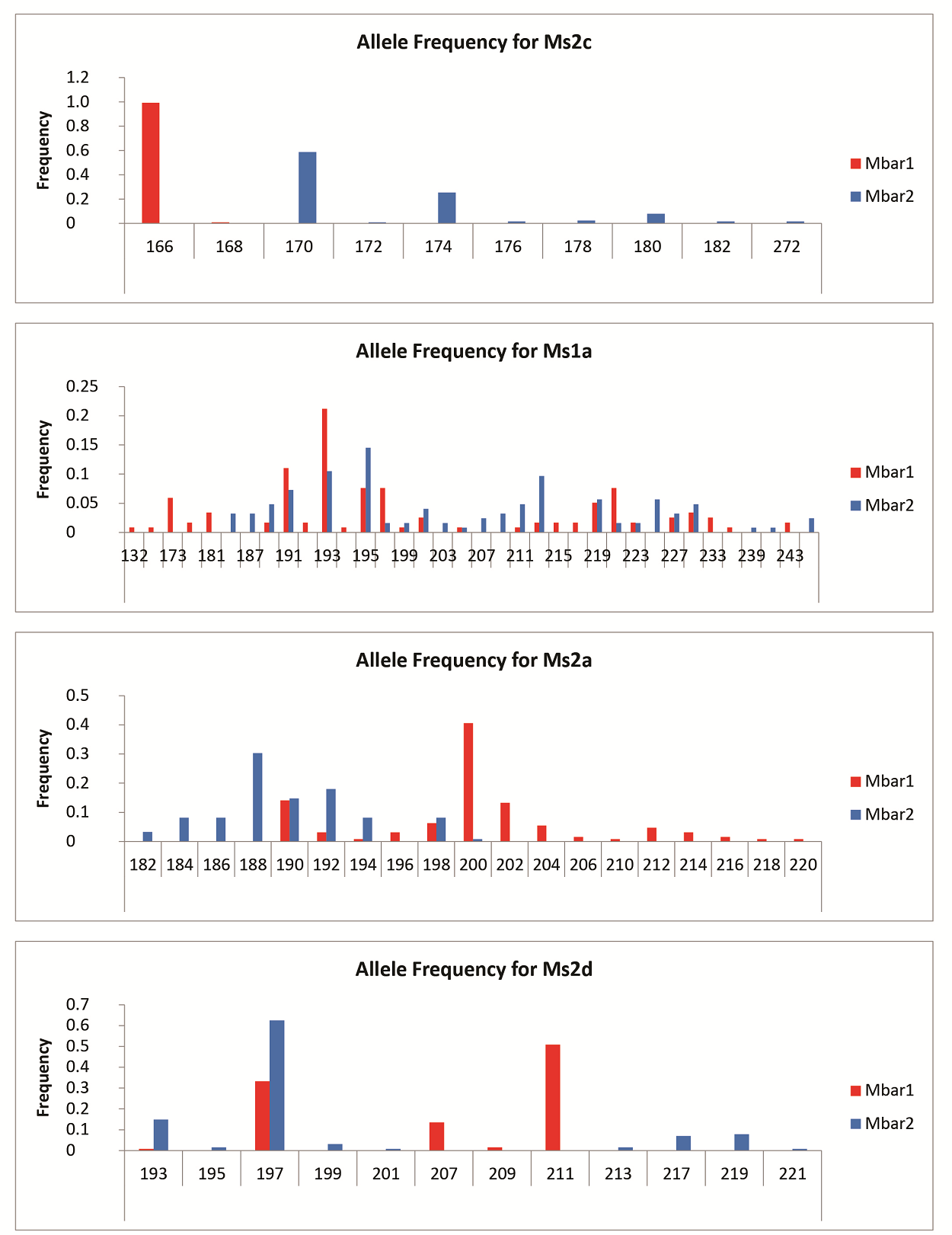

Supplement: Supplementary methods and results [file rsbl20160542supp1.docx]
